# Off-Label Yellow Fever and Hepatitis A Vaccination in Traveling Children

**DOI:** 10.3390/children11030374

**Published:** 2024-03-21

**Authors:** Cecilia Muruzábal, Lorea Vicente, Lucía Escolano Taravillo, Blanca Bravo Queipo de Llano, Cristina Calvo, Milagros García López Hortelano

**Affiliations:** 1 Pediatric and Infectious Diseases Department, La Paz University Hospital, 28046 Madrid, Spain; cecilia.muruzabal@salud.madrid.org (C.M.); lorea.vicente@salud.madrid.org (L.V.); lucia.escolano@salud.madrid.org (L.E.T.); blanca.bravo@salud.madrid.org (B.B.Q.d.L.); mghortelano@salud.madrid.org (M.G.L.H.); 2 La Paz Institute for Health Research (IdiPAZ), 28029 Madrid, Spain; 3 Biomedical Research Network Centre for Infectious Diseases (CIBERINFEC), Carlos III Health Institute, 28029 Madrid, Spain; 4 Translational Research Network in Pediatric Infectious Diseases (RITIP), 28046 Madrid, Spain; 5 Pediatric Tropical Diseases Unit, International Adoption and Consultation of Traveling Children, La Paz University Hospital, 28046 Madrid, Spain

**Keywords:** yellow fever, hepatitis A, vaccine, off-label, adverse effects

## Abstract

There are few data on yellow fever (YF) and hepatitis A (HA) off-label vaccination. Given the rising trend of travel to endemic countries, there is a growing necessity to broaden vaccination coverage among the pediatric population. For this reason, we aim to assess the adverse effects associated with off-label vaccination, with the ultimate purpose of expanding the vaccine spectrum. We analyzed ambispectively ninety-four children under 12 months of age who received YF or HA off-label vaccines. The YF vaccine was administered to children aged 6–9 months and those allergic to eggs (with a prior negative prick test and no history of anaphylaxis), while the HA vaccine was given to children aged 6–12 months. Overall, 71 (75%) were vaccinated against YF, and 57 (60%) against HA; 34 against both. All of them fulfilled off-label vaccination criteria. No immediate adverse effects (AEs) were reported. Mild common AEs (diarrhea, fever, or malaise) were experienced by 10.8% of patients within 10 days after vaccination. The rate of AEs associated with off-label vaccination for HA and YF is low, suggesting that the vaccines could be considered safe.

## 1. Introduction

Yellow fever (YF) and hepatitis A (HA) are vaccine-preventable viral diseases prevalent in tropical countries.

YF is transmitted by infected mosquitoes and affects densely populated areas with low vaccination rates. Most infected people develop a mild illness, but severe symptoms can occur in a small proportion of them, leading to high mortality rates. YF is endemic in 34 African and 13 Central/South American countries, causing an estimated 300,000 deaths annually [1].

The primary prevention strategy for YF is mosquito bite avoidance, but a safe and effective vaccine is available. Currently, a single dose of the vaccine is indicated, since in 2016 the requirement for a booster dose every 10 years was removed from the International Health Regulations [1]. However, some recent studies have evaluated long-term immunity following vaccine administration in children, registering lower seroconversion rates compared to the adult population [2,3]. These results cast doubt on the need for a booster dose in order to guarantee protective immunity in children, but to date, the vaccination schedule for this population group has not been modified.

The current vaccines employed for YF prevention are live attenuated virus vaccines. In Europe, the Stamaril^®^ vaccine is used, while in the United States, they use YF-VAX^®^. The virus used in the production of these vaccines is cultivated in chicken embryos, so the vaccine may contain traces of chicken egg protein (ovalbumin) [4]. No document has been published specifying the exact amount of ovalbumin contained in the vaccine, but most sources indicate that the concentration of this protein in the vaccine is extremely low.

YF vaccination is recommended for individuals ≥ 9 months old traveling to or residing in endemic areas. Contraindications includes severe hypersensitivity to the vaccine or its components and age <6 months, with limited approval for use in children aged 6–9 months and mild hypersensitivity reactions (such as an egg allergy without a history of anaphylaxis) [1].

On the other hand, hepatitis A virus (HAV) is primarily transmitted through contaminated water or food or direct contact with an infected person, and causes liver inflammation. HAV prevalence is higher in low/middle-income countries with inadequate sanitary conditions; therefore, the main prevention strategy for HA is the improvement of sanitation and food safety. Additionally, vaccination is an effective preventive measure and is indicated for individuals ≥12 months old traveling to HA-endemic areas [5].

There are two types of inactivated HA vaccines available for children (Havrix^®^ and Vaqta^®^), which are administered intramuscularly. The current vaccination schedule consists of two doses separated by at least 6 months; however, if the vaccine is administered under 12 months of age, two additional doses following the routine schedule are required [5]. Additionally, there is Twinrix^®^ (GSK, London, UK), a combined inactivated hepatitis A and B vaccine. Unlike HA vaccine, it requires three doses, at 0, 1, and 6 months, to acquire proper immunity.

Currently, there is limited information available regarding the off-label use of these vaccines in traveling children, which refers to its employment in an unapproved indication by the U.S. Food and Drug Administration (FDA) or in an unapproved age group, dosage, or route of administration. Therefore, this study aims to assess the immediate and delayed adverse effects of off-label YF and HA vaccines in the pediatric population within an international travel unit.

## 2. Materials and Methods

An ambispective review was conducted on children under 12 months of age who attended at the travelers’ consultation of an International Vaccination Center affiliated to La Paz University Hospital between 2018 and 2022, and who received YF or HA off-label vaccines. The study was approved by the Ethics Committee.

The inclusion criteria encompassed children vaccinated against YF between 6 and 9 months of age or those allergic to eggs, and children vaccinated against HA between 6 and 12 months of age. YF vaccine candidates with egg allergies underwent a skin prick test to assess their response before vaccination. Positive test results led to withholding vaccination, while negative results allowed vaccination.

Parents or guardians were provided with informed consent explaining potential AEs and given a contact number to report any observed AEs. YF vaccination involved a 0.5 mL subcutaneous dose of the Stamaril^®^ vaccine (Sanofi Pasteur, Gentilly, France), typically requiring no booster dose. HA vaccination required a 0.5 mL intramuscular dose. To achieve proper immunization, two additional doses were required at 12 months of age following the routine schedule. Havrix^®^ (GSK, London, UK) and Vaqta^®^ (MSD, Rahway, NJ, USA) vaccines were used interchangeably.

Four children under 9 months old, with the traveler condition of visiting friends and relatives (VFR), who traveled to the Amazon region of Brazil and Bolivia and to Sierra Leone, were categorized as very high-risk travelers according to the Centers for Disease Control and Prevention (CDC) Yellow Book classification. These children received both HA and YF vaccines [1].

Medical records were reviewed to collect epidemiological data, traveler type, travel duration, destination country, vaccine type administered, immediate AEs (within 30 min), and delayed AEs (up to 10 days after vaccination). Patients were contacted by phone for information on delayed AEs, and those unable to be reached were considered lost to the study.

## 3. Results

A total of 94 children were included in the study (of whom 57.9% were males), with a median age of 10 months (IQR 9–11) and a median weight of 9 kg (IQR 8.6–10.4). Forty-nine (52%) were vaccinated against YF and forty-five against HA (48%) with off-label indications. Thirty-four children (36%) received both vaccines simultaneously. Twenty-two children with an off-label indication for HA also received the YF vaccine, with an approved indication. These vaccination groups are shown in Table 1. Three children (3%) had mild pre-existing conditions (cardiac and neurological disease), that did not hinder their travel. A total of 14.9% (14/94) had an egg allergy, without a history of anaphylaxis, and with a negative result on the prick test, conducted prior to the administration of the vaccines.

The vaccination schedule was appropriate for their age in the majority (94/95). The most common reason for travel was VFR (88.4%) and the most frequent destination was Central America (74.7%). The median duration of travel was 30 days (IQR 21–60). In addition to the vaccines against YF and HA, five patients received vaccination against tetravalent meningitis (5%), three against measles, mumps, and rubella (MMR) (3%), and one against Japanese encephalitis (1%) at the Unit. Those patients vaccinated against MMR only received the HA vaccine, to avoid interference with the YF vaccine.

We did not observe any immediate adverse effects (AEs) in any of the patients within the first 30 min following vaccine administration. We were able to successfully contact by telephone 74 children. However, none of the 20 patients considered lost to follow up called the Unit because of any adverse effects. Of the 74 patients we reached, 8 (10.8%) reported having mild adverse effects, including the following: diarrhea 2/8 (25%), fever 5/8 (62.5%), and irritability 1/8 (12.5%), included in the technical data sheet. The duration of the fever was less than 24 h in all cases. All patients who experienced diarrhea had been vaccinated with the YF vaccine. Of the five patients who had fever, one (20%) had received the YF vaccine, three (60%) the HA vaccine, and one (20%) both vaccines simultaneously. Lastly, the one patient who experienced irritability had been vaccinated with both vaccines. All patients reported complete resolution of the described adverse event. No visits to the emergency room were recorded in any case. Results are presented in Table 1.

## 4. Discussion

Vaccination against YF and HA in travelers visiting endemic areas or at high risk of infection has led to a significant reduction in the prevalence of these diseases and, consequently, a decrease in mortality. Their use in the pediatric population is approved and it has been proved to be safe, but it is contraindicated in certain groups. The response in these groups is unknown, and therefore its use is not currently approved by the World Health Organization (WHO) or CDC [1]. However, both vaccines are necessary and particularly important in young children who are going to travel to endemic areas, and who are specifically vulnerable. For this reason, our study, carried out with adequate information for parents, and assessing the risks and benefits, provides valuable knowledge.

The adverse reactions to the YF vaccine are mostly mild, commonly fever, which may occur on the same day of administration or after 10–12 days. However, exceptionally, some serious adverse reactions have been described, such as hypersensitivity reactions, YF vaccine-associated neurologic disease (YEL-AND), and YF vaccine-associated viscerotropic disease (YEL-AVD), with minimal incidence according to the latest data published, and particularly higher in children <6 months [1]. Hypersensitivity reactions (rash, urticaria, or bronchospasm) are uncommon, and the incidence of anaphylactic reactions is exceptionally rare [1]. None of these adverse effects were reported in our sample.

Three cases of YEL-AND have been described in exclusively breastfed infants whose mothers had received the YF vaccine [1]. Until specific research data are available, the latest guidelines recommend avoiding YF vaccination in breastfeeding women except in situations where exposure to YF cannot be avoided or postponed. Some experts recommend that mothers should pump and discard breast milk at least 2 weeks after receiving the vaccine before restarting breastfeeding [6].

According to the technical data sheet, severe egg allergy is considered a contraindication for yellow fever vaccination, defined by most guidelines as a previous history of anaphylaxis. In our sample, these children were excluded from vaccination, as well as those with a positive vaccine prick test result. No adverse effects were recorded after vaccination, which allows us to presume that the vaccine is also safe in this group.

Other authors who have attempted to prove the safety of the YF vaccine in off-label population groups, discuss the necessity of conducting a prior prick test before administering the vaccine. Sharma et al. [7] demonstrated the absence of adverse effects after vaccine administration in a sample of 11 children allergic to egg, with 7 of them receiving a prior prick test, which yielded negative results. Additionally, they did not exclude from vaccination children with a history of egg anaphylaxis. In line with this approach, Cheung et al. [8] suggest that the prick test should be exclusively used in patients with a previous history of anaphylaxis due to the increased risk of severe adverse reactions in this group. They justify the safety of the YF vaccine in egg-allergic patients by stating that the amount of ovalbumin contained in the yellow fever vaccine is minimal and lower than the influenza vaccine, which is approved in our study groups.

Based on the study results and the previous bibliography, we can predict that the incidence of vaccine adverse effects in egg-allergic patients with a negative prick test is very low, supporting the approval of its use in this patient population and questioning the need for a prior vaccine prick test before its administration.

On the other hand, few data are available regarding the safety of the HA vaccine in children under 2 years old. The most frequent adverse effect associated with HA vaccination is soreness at the injection site, which is hard to assess in young children, and no serious adverse effects have been reported. Vaccination is not approved for children < 12 months of age, as it can interfere with pre-existing maternal antibodies [9]. Kanra et al. [10] conducted a clinical trial demonstrating seropositivity in 2-month-old children after receiving three doses of the HA vaccine, with only pain at the injection site and fever described as adverse effects.

According to our results, both vaccines could be safe in the off-label indications present in our series. However, we must acknowledge our limitations, such as the small sample size, that probably do not allow us to detect infrequent side effects, as well as the retrospective collection of delayed adverse effects and the loss to follow-up of some patients, which may have interfered with the results in the form of memory bias. Nevertheless, we provided patients with a contact telephone number, so we can presume that they did not have side effects.

Our results could be a starting point for new systematic studies on the off-label use of both vaccines in these children.

## Figures and Tables

**Table 1:** Off-label vaccination indications and frequency of vaccinated children. Immediate and delayed adverse effects following vaccine administration.

Off-label indications	*n* = 94
YF between 6 and 9 months of age	35 (37.2%)
YF egg-allergic patients (non-anaphylactic)	14 (14.9%)
HA <12 months of age	45 (47.9%)
Immediate adverse effects (first 30 min)	*n* = 94
Yes	0 (0%)
No	94 (100%)
Late adverse effects (first 10 days)	*n* = 94
Yes	8 (10.8%)
No	66 (89.2%)
* Loss to follow up	20 (21.3%)
Type late adverse effect	*n* = 8
Diarrhea	2 (25%)
YF	2 (100%)
Fever	5 (62.5%)
YF	1 (20%)
HA	3 (60%)
YF + HA	1 (20%)
Irritability	1 (12.5%)
YF + HA	1 (100%)

*n*: total number of patients, YF: yellow fever vaccine; HA: hepatitis A vaccine. * No telephone contact possible.

## Data Availability

The data presented in this study are available on request from the corresponding author. The data are not publicly available due to privacy concerns.
